# Vein Visualisation Technology for Peripheral Intravenous Access in Paediatric Patients: A Clinical Decision‐Making Tool

**DOI:** 10.1002/nop2.70054

**Published:** 2024-10-18

**Authors:** Elizabeth Weathers, Mary Cazzell, Julie A. Thompson, Kathy Grieser, Leticia Caraveo

**Affiliations:** ^1^ School of Nursing, Midwifery, and Health Sciences University College Dublin Belfield Ireland; ^2^ Cook Children's Medical Center Fort Worth Texas USA; ^3^ Duke University School of Nursing Durham North Carolina USA

**Keywords:** paediatric patients, peripheral intravenous access, PIV success rates, vascular access, vein visualisation technology

## Abstract

**Aim:**

The aim of this study is to develop a clinical decision‐making tool to guide utilisation of vein visualisation technologies and enhance chances of successful peripheral intravenous catheter (PIVC) insertion, using data collected from a vascular access team in a large paediatric medical centre in the United States.

**Design:**

Quantitative two‐phase, cluster analysis design.

**Methods:**

The study consisted of the following two phases: (1) a quantitative retrospective chart review to evaluate clinician utilisation and preference for vein visualisation technologies and (2) a quantitative prospective design, including a post‐discharge retrospective chart review, to confirm utilisation of vein visualisation technologies and factors influencing clinician decision‐making.

**Results:**

A total of 16 groups were created based on the cluster analysis and expert clinician input. The results of first‐attempt success analyses identified optimal device recommendations for each of the 16 patient groups. For patients older than 2 years old, the NIR device was more likely to result in first‐attempt success regardless of BMI or access site and the NIR device was most optimal for almost all categories of paediatric patients regardless of BMI or access site. The transilluminator was found to be the most optimal device to use with younger patients (< 2 years old) who are underweight.

**Conclusion:**

Vein visualisation technology is recommended by professional nursing organisations to improve PIV access. Yet, adoption of this useful technology to aid selection of an optimal vein for insertion and visualisation during insertion is limited. This is the first study to develop a clinical decision‐making tool for vein visualisation technology in PIVC insertion.

**Implications for the Profession and Patient Care:**

Vein visualisation technology allows for a rapid, thorough assessment of patients' vasculature to determine an optimal site for PIVC placement beyond what is visible to the naked eye or achievable using traditional methods. The tool was designed to guide healthcare professionals towards successful first attempt PIVC insertions, thereby improving patient outcomes and efficiency of care.

**Patient or Public Contribution:**

None.

## 1 Background and Significance

### 1.1 Complexity of the Procedure

More than 300 million peripheral intravenous catheters (PIVCs) are placed by healthcare providers annually, making it one of the most frequently performed procedures in the United States, with up to 90% of patients requiring a PIVC during their hospital admission (Alexandrou et al. 2018; Marsh et al. 2020). Despite being one of the most frequently performed invasive procedures in healthcare, PIVC placement often requires multiple attempts to place a single PIVC with high complication rates (Keleekai et al. 2016; van Loon et al. 2016; Rickard et al. 2018). The most common complications identified include phlebitis (15.4%–19.3%), infiltration or extravasation (13.7%–23.9%), partial or complete occlusion (8%–18.8%), leakage (7.3%), pain (6.4%), dislodgement (6%–6.9%), and infection (0.2%) (Marsh et al. 2020; Keleekai et al. 2016). Up to 90% of PIVCs are prematurely removed and require replacement due to failure or ineffective securement (Alexandrou et al. 2018; Rickard et al. 2018). Repeated attempts, or premature failure, lead to an increased use of resources, and an increased risk of venous depletion, nerve damage, paresthesia, hematomas, and arterial puncture (van Loon et al. 2016). Patient factors such as age, non‐palpable veins, skin tone, and body mass index (BMI), along with provider factors such as number of insertions performed, or pre‐insertion confidence, are all associated with odds of success (Carr et al. 2016).

### 1.2 Lack of Education and Experience

Frequently, PIVCs are placed by nurses (Alexandrou et al. 2018), many of whom are new graduates with limited experience (Keleekai et al. 2016). Some complications and premature failures can be attributed to operator error (Massey et al. 2020; Hawes 2007), since many nurses lack confidence in PIVC insertion (Lyons and Kasker 2012). Evidence suggests that there is variation in education and PIVC training in nursing programs across the United States (Keleekai et al. 2016; Lyons and Kasker 2012; Massey et al. 2020; Vizcarra et al. 2014; Brown, Crookes, and Iverson 2015). Up to 60% of nurses report not being taught how to insert a PIVC while in nursing school (Lyons and Kasker 2012; Vizcarra et al. 2014), creating a knowledge deficit in clinical practice (Etafa et al. 2020; Simonetti et al. 2019). First‐attempt success rates can range from 44% to 76.9% for junior staff nurses, compared with 91%–98% among experienced nurses (Keleekai et al. 2016). Multiple insertion attempts can lead to a myriad of complications including delay in treatment, patient pain and dissatisfaction, venous depletion, nerve injury, and vessel‐related trauma (van Loon et al. 2016; Parker et al. 2017; Helm et al. 2015). Increased PIVC placement attempts are also associated with an increased risk for needlestick injuries and potential transmission of a bloodborne pathogen (Reddy et al. 2017; Zhang, Lee, and Knott 2014; Pinelli and Pittiruti 2021). Repeated PIVC attempts lead to inefficient utilisation of staff and equipment resources (Goff et al. 2013). Evidence shows that patients who require greater than, or equal to, three attempts consume 43% of total PIVC costs (Hallam et al. 2016) and premature PIVC removal or failure is estimated to cost between $9.8 and $17.5 billion annually (Glover et al. 2017).

### 1.3 Improving PIVC Placement in the Paediatric Population

One of the most challenging populations for PIVC placement is paediatrics due to the depth of their peripheral veins (Park et al. 2016). First‐attempt success rates in children can range from 60% to 81% (Park et al. 2016). Several technologies are now available to assist and improve PIVC placement, including transilluminator, ultrasound, and near‐infrared (NIR) vein visualisation technology. The use of vein visualisation technology has been recommended by the Emergency Nurses' Association (ENA), the Association for Vascular Access (AVA), as well as the Infusion Nurses Society (INS) (Parker et al. 2017; Nickel et al. 2024; Pitts and Ostroff 2020). Vein visualisation technology enhances users' skills by allowing them to thoroughly assess patient anatomy and vasculature and more efficiently determine an optimal site for PIVC placement beyond what is visible to the naked eye or achievable using traditional methods (anatomical landmarks). Optimising site selection by identifying areas away from flexion points (e.g., the antecubital fossa) and above the wrist, and away from valves or bifurcations, is not only more comfortable for patients, but results in improved patency of the catheter, reduced need to replace PIVCs, and increased patient satisfaction (Çağlar et al. 2019; Eren and Caliskan 2021). NIR enables accurate visualisation of subcutaneous vasculatures up to 10 mm deep. NIR can be differentiated from other technologies because it allows visualisation of multiple vessels at one time as opposed to visualising one vein at a time as with other devices (e.g., transilluminator, ultrasound).

The use of vein visualisation technologies has been assessed in the paediatric population. Inconsistencies exist in the literature, despite the publication of several meta‐analyses and systematic reviews (Park et al. 2016; Kuo, Feng, and Lee 2017; Parker, Benzies, and Hayden 2017). Overall, most studies reported first‐attempt success rates ranging from 47%–79% for traditional techniques compared with 62%–79% using NIR (*p* = 0.361–0.085) (Çağlar et al. 2019; Perry, Caviness, and Hsu 2011; Cuper et al. 2013; Chapman et al. 2011; van der Woude et al. 2013; Curtis et al. 2015; Szmuk et al. 2013; de Graaff et al. 2014; Rothbart et al. 2015; Kim et al. 2012; Guillon et al. 2015; Kaddoum et al. 2012). Regarding time to PIVC placement, an average of 53–246 s in the vein visualisation group has been reported versus 68–300 s in the control group (*p* = 0.10–0.54) (Helm et al. 2015; Zhang, Lee, and Knott 2014; Hallam et al. 2016; Glover et al. 2017; Pitts and Ostroff 2020). Yet, no statistically significant improvement was found for time to cannulation or first‐attempt success when using vein visualisation technology (Cuper et al. 2013; van der Woude et al. 2013; Curtis et al. 2015; de Graaff et al. 2014; Rothbart et al. 2015; Kaddoum et al. 2012). Studies have also investigated clinician‐user, patients, and family ratings of vein visualisation technology and results showed that the technology is perceived as helpful and of benefit to patient care, including a reduction in patient pain and costs (Perry, Caviness, and Hsu 2011; Chapman et al. 2011; Guillon et al. 2015; Sun et al. 2013).

In summary, vein visualisation technology appears to be well received and positively evaluated by clinicians, patients, and family members. Furthermore, vein visualisation technology is recommended by several professional organisations as a means of improving PIVC placement. Yet, adoption of this useful technology to aid site assessment, selection of an optimal vein for insertion, and visualisation during insertion, is limited. In the context of paediatric care, PIVC placement can be an especially traumatic experience, which underscores the need to implement measures that enhance the chances of success with PIVC placement. Therefore, this study sought to develop a clinical decision‐making tool to guide utilisation of vein visualisation technologies, including NIR and transilluminator.

### 1.4 Purpose/Aims

The primary aim was to develop a clinical decision‐making tool for successful PIVC insertion based on data collected from a vascular access team (VAT) in a large paediatric medical centre in the southwestern United States. The study consisted of two phases: phase 1 was a retrospective study to investigate clinician utilisation and preference for vein visualisation technologies; and phase 2 was a prospective study to further analyse utilisation of vein visualisation technologies and factors influencing clinician decision‐making.

## 2 Methods

### 2.1 Study Design

This study utilised: (1) a quantitative retrospective chart review design to evaluate utilisation and preference for different vein visualisation technologies based on data from the VAT staff and (2) a quantitative prospective design whereby VAT staff documented, in real‐time, PIVC access data (device, number of attempts, diagnosis) in addition to a post‐discharge retrospective chart review to obtain additional study data from the electronic medical record (EMR). Approval was received from the Institutional Review Board at the paediatric medical centre. A waiver of informed consent and a waiver of authorization for the use and/or disclosure of protected health information were granted.

### 2.2 Setting and Sample

This study was conducted at a large tertiary care paediatric medical centre in Texas, USA. The medical centre includes 444 licensed beds and approximately 1500 nurses. The VAT staff consisted of one Nurse Manager, 12 Registered Nurses, and four paramedics. The VAT start PIVCs, troubleshoot IVs, evaluate infiltrations and extravasations, treat extravasations that meet criteria for treatment, place midlines and central lines, restore patency to central lines, repair central lines, and draw labs on patients deemed “difficult sticks” in the outpatient lab, on units, and in the Emergency Department. During September 2022 and upon completion of phase 2, the VAT staff started a total of 1149 PIVCs with a daily average of 40 PIVC insertions.

All paediatric patients (birth to 21 years) who received at least one PIVC placement attempt from the VAT staff and were admitted within inpatient areas (critical care units, medical/surgical units, sub‐specialty units, or emergency services) were eligible for study inclusion. In phase 1, from October through December 2021, retrospective data were collected on 500 documented PIVC insertions by VAT staff. For phase 2, from July through August 2022, prospective data, on 100 PIV insertions, were collected by VAT staff when called to start PIVCs. Post‐discharge retrospective EMR data were collected by members of the research team on these same PIVC attempts/insertions.

### 2.3 Data Collection

#### 2.3.1 Phase 1 Retrospective Data

The study team utilised an already‐existing quality data dashboard created by a medical centre Business Intelligence analyst based on PIVC insertions/attempts by VAT staff. This data included: (1) patient characteristics: age, gender BMI, and race/skin tone; (2), category of primary admitting diagnoses; and (3) PIVC removal information related to dwell time (length of time PIVC remained in place).

#### 2.3.2 Phase 2 Prospective Data

The Prospective Data Collection Sheet was created based on a literature review and the VAT clinical expertise. Prospective data collection included: (1) first‐attempt success (yes/no): describing ability of VAT staff to cannulate a peripheral vein on first attempt; (2) estimated Difficulty of IV Access (DIVA) score (includes vein visible and palpable after tourniquet, age, and history of difficult access); (3) number of unsuccessful PIV attempts before VAT arrival (how many and by whom); (4) number of overall PIVC attempts by VAT staff; (5) additional data for up to five PIV attempts (success or if care escalated); (6) peripheral intravenous (PIV) characteristics to describe existing PIV cannulation practices: catheter size, orientation (right/left), access site, catheter type; and (7) type of assist device or traditional technique used per PIVC attempt (NIR, transilluminator, ultrasound, or anatomical landmarks). Post‐discharge retrospective data on the 100 PIVC attempts included: (1) patient characteristics: age, gender BMI, and race/skin tone; (2), category of primary admitting diagnoses; (3) unit where patient was admitted; (4) PIVC removal information related to dwell time (length of time PIVC remained in place) and reason for removal; and (5) list of IV medications administered.

### 2.4 Statistical Analysis

For phase 1, a two‐step cluster analysis using log likelihood distance measuring, was conducted to investigate clinician utilisation and preference for vein visualisation technologies. Continuous variables included age and BMI. Categorical variables included skin tone and device. The number of clusters was set to a maximum of 15. Groups based on patient demographics (age, BMI, gender, and race/skin tone) were developed in relation to proportion of attempts by each device.

Phase 2 consisted of consultation with three clinical experts to derive patient groups and determine the association between patient group assignment and optimal device for first attempt PIVC success. Data‐based groups from the cluster analysis findings in phase 1 and clinical insight from phase 2 interviews with experts were utilised to assign patients to groups based on clinical factors (e.g., access site) and demographic factors (e.g., age, BMI). Chi‐square tests were performed to determine the impact of device on first attempt success for each patient group, separately. Data were analysed in IBM SPSS version 29 (Armonk, NY). The level of significance was set to 0.05.

## 3 Results/Findings

For phase 1, the two‐step cluster analysis showed an association between age and BMI for clinician technology preferences. A total of three clusters were identified with a Silhouette measure of cohesion and separation = 0.45. Gender and skin tone were distributed in near‐equal proportions among all technologies, and thus, were not considered differentiating factors in clinician technology preference. Ultrasound was utilised for just three clinical cases in the reported data and therefore, was removed from further analysis. Table 1 displays the findings for the clusters.

**TABLE 1:** Phase 1 Two‐step cluster findings.

Clinician technology preference	Age, mean (SD)	Skin tone %	BMI mean (SD)	Gender %
Transilluminator *N* = 99	1.66 (3.10)	Dark: 55% Light: 45%	16 (2.0)	Male: 46% Female: 54%
Near infrared *N* = 177	7.9 (6.5)	Dark: 58% Light: 42%	20 (6.1)	Male: 49% Female: 51%
Anatomical landmarks (Traditional method) *N* = 109	9.1 (5.7)	Dark: 54% Light: 46%	18 (4.5)	Male: 53% Female: 47%

Based on the findings from phase 1, three clinicians were consulted for confirmation of findings and to identify other factors for consideration in a PIVC clinical decision‐making tool. Preferences for age and BMI were confirmed, in addition to acknowledgement that gender and race/skin tone were not primary factors in technology preference. Based on the empirical findings and clinical expert assessments of phase 1, phase 2 analyses focused on associations between age, BMI, access site, and optimal technology for PIVC first‐attempt success.

From the total 600 retrospective and prospective PIVC attempt records collected, completed data were available for 585 patients and were included in the analyses. For the 15 entries with missing data, three were missing access sites, eight were missing BMI, and four were missing age. Patient demographic and clinical characteristics of gender, age, BMI, and access site are presented in Table 2.

**TABLE 2:** Patient demographics and clinical characteristics.

Variable	*n*	%
Gender		
Male	274	46.8
Female	311	53.2
Age		
< 2	222	37.9
2–5	92	15.7
6–12	99	16.9
Older than 12	172	29.4
BMI		
Underweight	362	61.9
Normal weight	152	26.0
Overweight or obese	71	12.1
Access site		
Hand or wrist	257	43.9
Forearm	196	33.5
AC or upper arm	16	2.7
Foot or saphenous	116	19.8

*Note:* % may not equal 100 due to rounding.

Patients were assigned to groups based on the phase 1 cluster analysis findings in conjunction with expert clinicians' input on access site. Gender and race/skin tone were omitted from the clinical decision‐making tool analysis based on empirical findings showing equal proportions of technology utilisation regardless of gender and skin tone and confirmation from clinician experts that these two demographic characteristics were not primary factors in clinician preference. For phase 2, a total of 16 groups were created as a result of statistical findings in phase 1 and clinician input leading to phase 2 (see Table 3).

**TABLE 3:** Empirically and clinically derived patient groups.

Group	*n*	Age category	BMI category	Access site
1	79	Younger than two	Underweight	Hand/Wrist
2	33	Younger than two	Underweight	Forearm/Upper arm
3	71	Younger than two	Underweight	Foot/Saphenous
4	21	Younger than two	Normal weight	Hand/Wrist
5	18	Younger than two	Normal weight	Forearm/Upper arm
6	45	Ages 2–5	Underweight	Hand/Wrist
7	28	Ages 2–5	Underweight	Forearm/Upper arm
8	19	Ages 2–5	Normal weight	Hand wrist or arm
9	31	6–12	Underweight	Hand/Wrist
10	40	6–12	Underweight	Forearm/Upper arm
11	41	6–12	Normal weight/Overweight	Hand/Wrist or Arm/Foot
12	35	Older than 12	Underweight	Hand/Wrist
13	30	Older than 12	Normal weight	Hand/Wrist
14	39	Older than 12	Normal weight	Forearm/Upper arm
15	26	Older than 12	Overweight	Hand/Wrist
16	29	Older than 12	Overweight	Forearm/Upper arm or Foot/Saphenous

Table 4 displays the statistical results of the first‐attempt success analyses and optimal device by patient group using the categories from the clinical decision‐making tool. As shown, the transilluminator is the most optimal device to use for PIVC attempts for patients < 2 years old if the patient is underweight. For patients < 2 years old and normal weight, the transilluminator or the NIR device are most optimal. For patients older than 2 years old, the NIR device was more likely to result in first‐attempt success regardless of BMI or access site. Statistical and clinical significance were considerations for developing the decision tool based on *p* < 0.05 and *p* < 0.10, respectively.

**TABLE 4:** Optimal device by group.

Group	Categories from clinical decision‐making tool	Optimal device^a^
1	Younger than two: Underweight	Transilluminator^b^
2	Younger than two: Underweight	Transilluminator^b^
3	Younger than two: Underweight	Transilluminator^c^
4	Younger than two: Normal weight	Transilluminator^c^
5	Younger than two: Normal weight	Near infrared^c^
6	Ages 2–5: Underweight	Transilluminator or near infrared^c^
7	Ages 2–5: Underweight	Near infrared^c^
8	Ages 2–5: Normal weight	Near infrared^b^
9	Ages 6–12: Underweight	Near infrared^b^
10	Ages 6–12: Underweight	Near infrared^c^
11	Ages 6–12: Normal weight or overweight	Near infrared
12	Older than 12: Underweight	Near infrared^c^
13	Older than 12: Normal weight	Near infrared^c^
14	Older than 12: Normal weight	Near infrared^c^
15	Older than 12: Overweight or obese	Near infrared
16	Older than 12: Overweight or obese	Near infrared

*Note:* If no symbol is presented, the device listed had the highest success rate, but the *p* > 0.10.

^a^ Optimal device in terms of likelihood of first attempt success.

^b^ Indicates statistical significance (*p* < 0.05).

^c^ Indicates clinical significance (*p* < 0.10).

**FIGURE 1:**
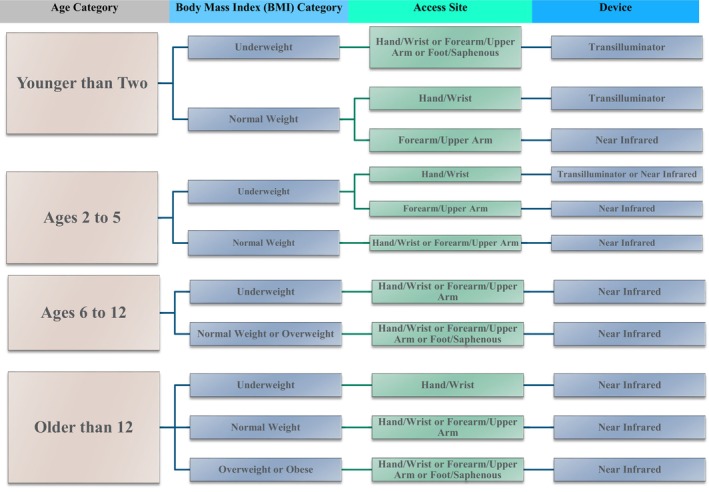
Vein visualisation clinical decision‐making (VV‐CDM) tool.

A chi‐square test was conducted for each patient group to compare first‐attempt success rates between devices (NIR and transilluminator) and to determine the most optimal device according to age, BMI, and access site. A preliminary clinical decision‐making tool for device recommendations was developed based on the results (see Figure 1: VV‐CDM).

## 4 Discussion

From two phases of data collection by VAT staff, the study results led to the first‐ever development of a vein visualisation clinical decision‐making tool for successful PIVC first‐attempt access in paediatric patients. Age (younger than 2 years old, 2–5 years old, 6–12 years old, and older than 12 years old), BMI (underweight, normal weight, overweight/obese) and access sites statistically and clinically predicted the type of assist device to use (NIR or transilluminator). Gender, race/skin tone, primary diagnosis, and DIVA score did not contribute to PIVC first‐attempt success.

### 4.1 Use of NIR Assist Device for PIVC Insertions

The Vein Visualisation Clinical Decision‐Making Tool (VV‐CDM) highlights the use of NIR as most optimal for almost all categories of paediatric patients regardless of BMI or access site. In patients younger than 2, the VV‐CDM emphasizes the use of NIR as most optimal for those with a normal BMI and when trying to access the forearm/upper arm (see Figure 1). To address conflicting research results on effectiveness of NIR on first‐time success rates, number of attempts, and procedure time (Kim et al. 2012; Kaddoum et al. 2012; Heinrichs et al. 2013; Demir and Inal 2019; Kleidon et al. 2021), a 2022 meta‐analysis was conducted on seven eligible studies (Feng et al. 2022). First‐time success rates were significantly improved (*p* = 0.03) for those with high DIVA scores when NIR was used but not for low to moderate DIVA scores, compared with traditional methods (Feng et al. 2022). The 2022 meta‐analysis also suggested that the use of NIR significantly decreased number of attempts (*p* < 0.0001) and procedure time (*p* < 0.0001) compared with traditional methods (Feng et al. 2022). This 2‐phase study's statistical analyses did not find DIVA score to be a predictor for first‐time success; however, age is included in DIVA scores; and BMI may affect the visibility and palpability of vessels.

### 4.2 Use of Transilluminator Assist Device for PIVC Insertions

The VV‐CDM highlights the transilluminator as most optimal for paediatric patients younger than 2 who are underweight regardless of access site or of normal weight when accessing hand, arm, or foot veins. For patients 2–5 years old who are underweight, the transilluminator is most optimal for accessing hand or wrist veins (see Figure 1). Two studies (Hosokawa et al. 2010; Dutt et al. 2023) and one systematic review (Firooz, Karkhah, and Hosseini 2022) reported significantly higher first‐attempt success rates with transilluminators in children less than 2 years old compared with anatomical landmarks. More research is needed to further validate this clinical decision‐making tool for use of transilluminator with children older than 2 years old.

### 4.3 Importance of VAT on Paediatric Atraumatic Care

In this study, the PIVC attempts were completed by VAT staff, who are a specialised proficient team, trained in the use of multiple assist devices, with higher PIVC access success, less IV complications, improved patient/family satisfaction, and above all, reduced pain and anxiety during an invasive traumatic procedure (Barreras and Chang 2017). One paediatric facility, without VAT resource, conducted a non‐blinded randomised control study to evaluate the introduction of a NIR device on paediatric nurses' perceived levels of skill and confidence in PIVC attempts (McNeely et al. 2018). No differences were noted between nurses in either group in terms of perceived skills or confidence with PIVC placement (McNeely et al. 2018). However, the sample was small (*n* = 27) limiting generalizability and the authors indicated that participants were experienced nurses. Prior research shows that clinician experience and uninterrupted skill practice contribute to 98.1% PIVC success rates compared with 77.6% success rates for inexperienced staff (Chu et al. 2023). Frontline nurses, may struggle to stay skilled and confident with PIVC placement due to their patient care obligations. Thus, specialised VATs in paediatric facilities can help promote atraumatic care (Öntürk et al. 2023). Furthermore, the use of the VV‐CDM tool and vein visualization technologies can enhance the confidence of frontline nurses and promote the delivery of atraumatic care.

## 5 Study Limitations

Two of the clinical groups had small sample sizes (< 20), which may limit generalizability of the VV‐CDM tool. Predictive modelling using larger samples will confirm this correlative‐ and evidence‐based clinical decision‐making tool. This study analysed data from PIVC attempts by VAT staff, from one paediatric healthcare facility, who are recognised for their clinical expertise. Not all healthcare facilities have VATs, so further testing of the VV‐CDM with frontline clinical non‐VAT nurses is warranted. It would be useful to repeat the data collection at different time periods, with teams from various units (e.g., medical‐surgical staff, oncology staff, etc.).

## 6 Implications for Future Research

Future studies should examine the VV‐CDM for use with frontline paediatric nurses as well as skilled VAT staff, and consideration should be given to the impact of age, BMI, and access site when choosing a vein visualisation device for PIVC placement. The VV‐CDM can be used in implementation studies to not only identify the predictive value for first attempt PIVC success but also examine the long‐term success, such as dwell time (how long the IV remained patent) (Hartman et al. 2020).

## 7 Linking Evidence to Action

- Vein visualisation technology is recommended by the ENA, AVA, and INS—it enhances users' skills by allowing them to thoroughly assess patients' superficial vasculature and more efficiently determine an optimal site for PIVC placement beyond what is visible to the naked eye or achievable using traditional methods (anatomical landmarks).
- This is the first study to develop a clinical decision‐making tool for utilisation of vein visualisation technology in PIVC insertion. The tool was designed to guide healthcare professionals towards successful first attempt PIVC insertions, thereby improving patient outcomes and care efficiency.
- This clinical decision‐making tool can be used as part of quality improvement initiatives that seek to improve alignment with the INS Standards of Practice and implement vein visualisation technology as a routine step for PIVC insertion procedures.

## 8 Conclusions

The VV‐CDM tool was created to guide healthcare professionals towards successful first‐time PIVC insertions through consideration of age, BMI, access site, and appropriate type of assist device. In paediatrics, the ultimate patient care goal is family‐centred atraumatic care; this clinical decision‐making tool could support clinicians in implementing best practices for PIV access.

## Author Contributions

Study design: E.W., M.C., J.A.T. Ethical approval: M.C., K.G., L.C. Data collection: M.C., K.G., L.C. Data analysis: J.A.T. Write‐up: E.W., M.C., J.A.T., K.G., L.C.

## Conflicts of Interest

The authors would like to disclose the following potential conflicts of interest: (1) Elizabeth Weathers was formerly employed as the Director of Medical and Clinical Affairs with AccuVein Inc., a company that specializes in vein visualization technology and (2) Julie Thompson provides ad‐hoc statistical consultancy services for AccuVein Inc. Every effort has been made to ensure that the research and findings presented in this manuscript are objective and unbiased, and the authors have taken measures to mitigate any influence that this affiliation may have had on the design, conduct, analysis, and reporting of the research. The data and conclusions presented in this manuscript are based on thorough and rigorous research procedures. This was a collaborative study in which all authors had input and oversight of every step of the research study including study design, IRB application and approval, data collection and statistical analysis, write‐up, and dissemination. We are committed to transparency and the integrity of the research process.

## Data Availability

The data that support the findings of this study are available on request from the corresponding author. The data are not publicly available due to privacy or ethical restrictions.
